# Dashboard With Bump Charts to Visualize the Changes in the Rankings of Leading Causes of Death According to Two Lists: National Population-Based Time-Series Cross-Sectional Study

**DOI:** 10.2196/42149

**Published:** 2023-06-27

**Authors:** Shu-Yu Tai, Ying-Chen Chi, Yu-Wen Chien, Ichiro Kawachi, Tsung-Hsueh Lu

**Affiliations:** 1 Department of Family Medicine Kaohsiung Municipal Ta-Tung Hospital and Kaohsiung Medical University Hospital; School of Medicine, College of Medicine Kaohsiung Medical University Kaohsiung Taiwan; 2 Department of Healthcare Information and Management School of Health Technology Ming Chuan University Taoyuan Taiwan; 3 Department of Public Health College of Medicine National Cheng Kung University Tainan Taiwan; 4 Department of Social and Behavioral Sciences Harvard T.H. Chan School of Public Health Boston, MA United States

**Keywords:** COVID-19, dashboard, data visualization, leading causes of death, mortality/trend, ranking, surveillance, cause of mortality, cause of death, monitoring, surveillance indicator, health statistics, mortality data

## Abstract

**Background:**

Health advocates and the media often use the rankings of the leading causes of death (CODs) to draw attention to health issues with relatively high mortality burdens in a population. The National Center for Health Statistics (NCHS) publishes “Deaths: leading causes” annually. The ranking list used by the NCHS and statistical offices in several countries includes broad categories such as cancer, heart disease, and accidents. However, the list used by the World Health Organization (WHO) subdivides broad categories (17 for cancer, 8 for heart disease, and 6 for accidents) and classifies Alzheimer disease and related dementias and hypertensive diseases more comprehensively compared to the NCHS list. Regarding the data visualization of the rankings of leading CODs, the bar chart is the most commonly used graph; nevertheless, bar charts may not effectively reveal the changes in the rankings over time.

**Objective:**

The aim of this study is to use a dashboard with bump charts to visualize the changes in the rankings of the leading CODs in the United States by sex and age from 1999 to 2021, according to 2 lists (NCHS vs WHO).

**Methods:**

Data on the number of deaths in each category from each list for each year were obtained from the Wide-ranging Online Data for Epidemiologic Research system, maintained by the Center for Disease Control and Prevention. Rankings were based on the absolute number of deaths. The dashboard enables users to filter by list (NCHS or WHO) and demographic characteristics (sex and age) and highlight a particular COD.

**Results:**

Several CODs that were only on the WHO list, including brain, breast, colon, hematopoietic, lung, pancreas, prostate, and uterus cancer (all classified as cancer on the NCHS list); unintentional transport injury; poisoning; drowning; and falls (all classified as accidents on the NCHS list), were among the 10 leading CODs in several sex and age subgroups. In contrast, several CODs that appeared among the 10 leading CODs according to the NCHS list, such as pneumonia, kidney disease, cirrhosis, and sepsis, were excluded from the 10 leading CODs if the WHO list was used. The rank of Alzheimer disease and related dementias and hypertensive diseases according to the WHO list was higher than their ranks according to the NCHS list. A marked increase in the ranking of unintentional poisoning among men aged 45-64 years was noted from 2008 to 2021.

**Conclusions:**

A dashboard with bump charts can be used to improve the visualization of the changes in the rankings of leading CODs according to the WHO and NCHS lists as well as demographic characteristics; the visualization can help users make informed decisions regarding the most appropriate ranking list for their needs.

## Introduction

Vital statistics can be used to monitor the vital signs of a nation [1]. Health advocates and the media often use the rankings of the leading causes of death (CODs) to draw attention to major health issues with relatively high mortality burdens in a population. The National Center for Health Statistics (NCHS) publishes “Deaths: leading causes” annually [2]. However, the ranking list used by the NCHS and statistical offices in several countries includes broad categories such as cancer, heart disease, and accidents [3-7]. The International Classification of Diseases and Health Related Problem Tenth Revision (ICD-10) has 89 subcategories in the broad cancer category, 41 subcategories in the broad heart disease category, and more than 100 subcategories in the broad accidents category [8]. Different subcategories within a broad category may have different levels of preventability, and therefore, may require different prevention strategies. For example, the 2019 version of the Organisation for Economic Co-operation and Development/Eurostat list of preventable and treatable CODs includes only 16 out of 89 cancer sites [9]. Stakeholders in public health policy require more specific and actionable information to help them set priorities.

To address the limitations of using broad categories for ranking, the World Health Organization (WHO) proposed a list in which cancer was divided into 17 cancer sites, heart disease into 8 specific forms of heart disease, and accidents into 6 unintentional injuries. Furthermore, more comprehensive COD categories are defined in the WHO list. For example, in the WHO list, the Alzheimer disease and related dementias (ADRD) category has ICD-10 codes F01 (vascular dementia), F03 (unspecified dementia), and G30 (Alzheimer disease), whereas in the NCHS list, ICD-10 code G30 is the only code available to classify ADRD-related mortality. In the WHO list, the hypertensive diseases category includes ICD-10 codes I10 (essential [primary] hypertension), I11 (hypertensive heart disease), I12 (hypertensive renal disease), I13 (hypertensive heart and renal disease), and I15 (secondary hypertension); however, in the NCHS list, ICD-10 codes I10, I12, and I15 are composed as one rankable category (essential hypertension and hypertensive renal disease) and I11 as well as I13 are included in the broad heart disease category. The WHO list has been routinely used to present the leading CODs by the WHO, the United Kingdom, Australia, and Italy [10-13]. However, the ranking profiles of leading CODs in the United States according to the WHO list remain unexplored.

Regarding the data visualization of the rankings of the leading CODs, the bar chart is the most commonly used chart [10-12,14]. However, it is difficult to reveal the changes in the rankings over time through bar charts. For example, the stacked bar chart by year was used by the UK Office for National Statistics; nevertheless, it was difficult to detect the changes in the ranking of a particular COD across years [11]. Bump charts have been recommended to represent the changes in the position of a given number of competing entities over a fixed duration [15]. Accordingly, in this study, we sought to design a dashboard with bump charts to visualize the changes in the rankings of the leading CODs in the Unites States by sex and age from 1999 to 2021 according to 2 lists (NCHS vs WHO).

## Methods

The data on the number of deaths in each category were obtained from the Wide-ranging Online Data for Epidemiologic Research system maintained by the Center for Disease Control and Prevention for the period from 1999 to 2021 [16]. The NCHS list had 52 categories, and the WHO list had 65 categories. The name and ICD-10 codes of each category in the WHO list mapped to the NCHS list are presented in Table S1 in Multimedia Appendix 1. We followed the method used by the NCHS to determine the rank of each category. The NCHS determines the rank of each category on the basis of the number of deaths in each category.

We used Tableau to create a dashboard with a bump chart that can be filtered by list and demographic characteristics (ie, sex and age), which allows the viewers to select the dimension in which they are interested [15]. In the bump chart, each COD has a specific color, and the abbreviated name of each COD category appears in each circle. When the cursor points to a circle in the bump chart, a tooltip appears and indicates the number of deaths for that category, the proportion of total deaths that category accounts for, and the full name of the category. We also designed a filter to highlight the particular COD.

### Ethical Considerations

This study used publicly available mortality data. All data used in this study were anonymized. This study was approved by the Institutional Review Board of National Cheng Kung University Hospital (B-EX-112-016) and the requirement of informed consent was waived by the abovementioned ethics committee.

## Results

**Figure 1:**
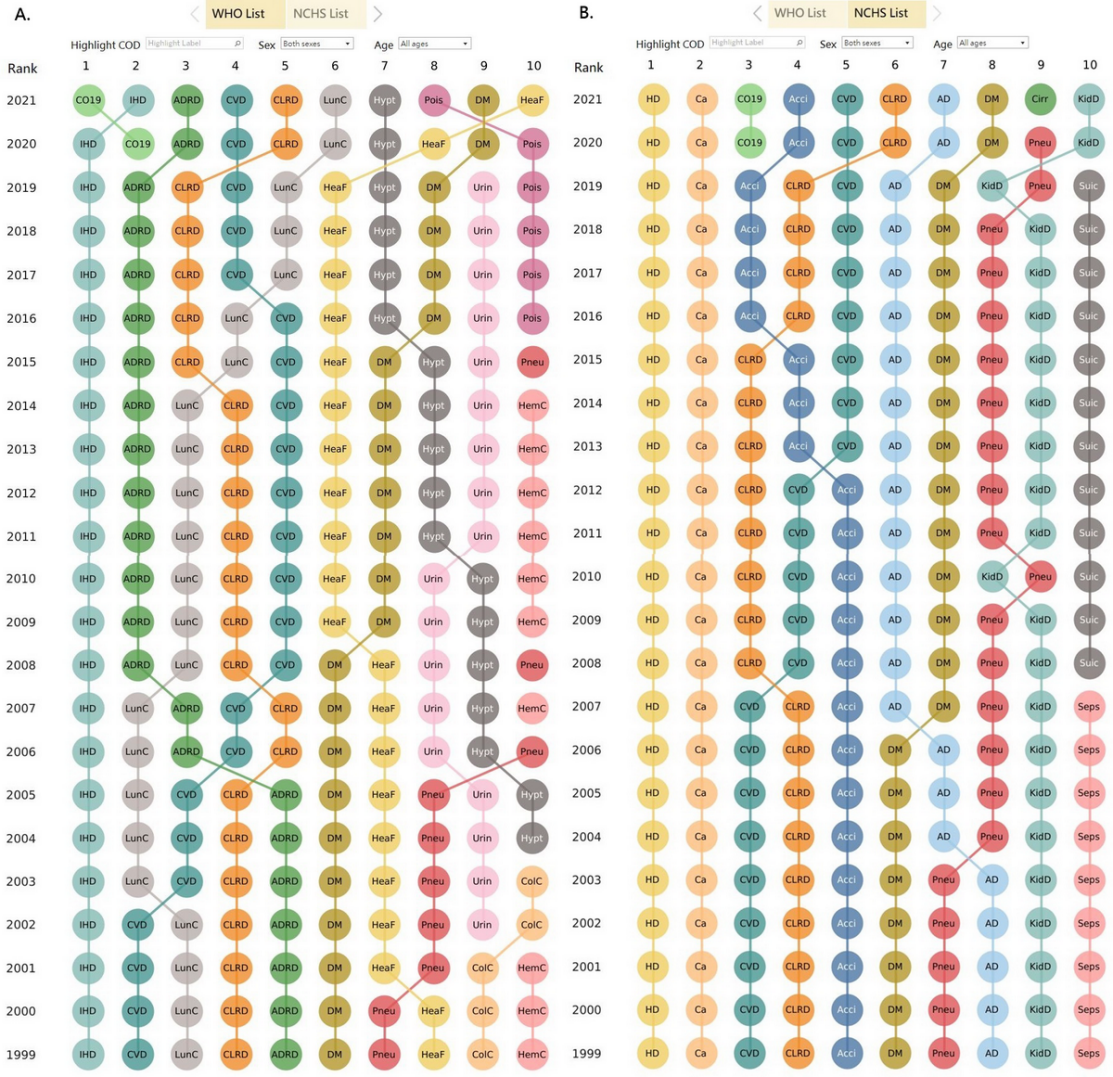
Ranking of the 10 leading causes of death for both sexes of all ages in the United States according to (A) the World Health Organization (WHO) list and (B) the National Center for Health Statistics (NHCS) list. This dashboard is accessible [17]. Acci: accidents; AD: Alzheimer disease; ADRD: Alzheimer disease and related dementias; Ca: cancer; Cirr: chronic liver disease and cirrhosis; CLRD: chronic lower respiratory disease; CO19: COVID-19; CVD: cerebrovascular disease (stroke); DM: diabetes mellitus; HD: heart disease; HeaF: heart failure; HemC: hematopoietic cancer; Hypt: hypertensive diseases; IHD: ischemic heart disease; KidD: kidney diseases; LunC: lung cancer; Pneu: pneumonia; Pois: unintentional poisoning; Seps: sepsis; Suic: suicide; Urin: urinary diseases.

According to the NCHS list, the first and second leading CODs for both sexes of all ages were heart disease and cancer throughout the 23-year study period (Figure 1B). However, according to the WHO list, the first and second leading CODs were ischemic heart disease (IHD) and cerebrovascular disease from 1999 to 2002, IHD and lung cancer from 2003 to 2007, IHD and ADRD from 2008 to 2019, IHD and COVID-19 in 2020, and COVID-19 and IHD in 2021 (Figure 1A). In the WHO list, IHD and heart failure were each identified in the 10 leading CODs, whereas in the NCHS list, these 2 conditions were both classified as heart diseases and are not distinguished. Likewise, lung cancer and hematopoietic cancer were among the 10 leading CODs in the WHO list but were not distinguished in the NCHS list because in the NCHS list they were both classified into the broader cancer category (Figure 1A).

**Figure 2:**
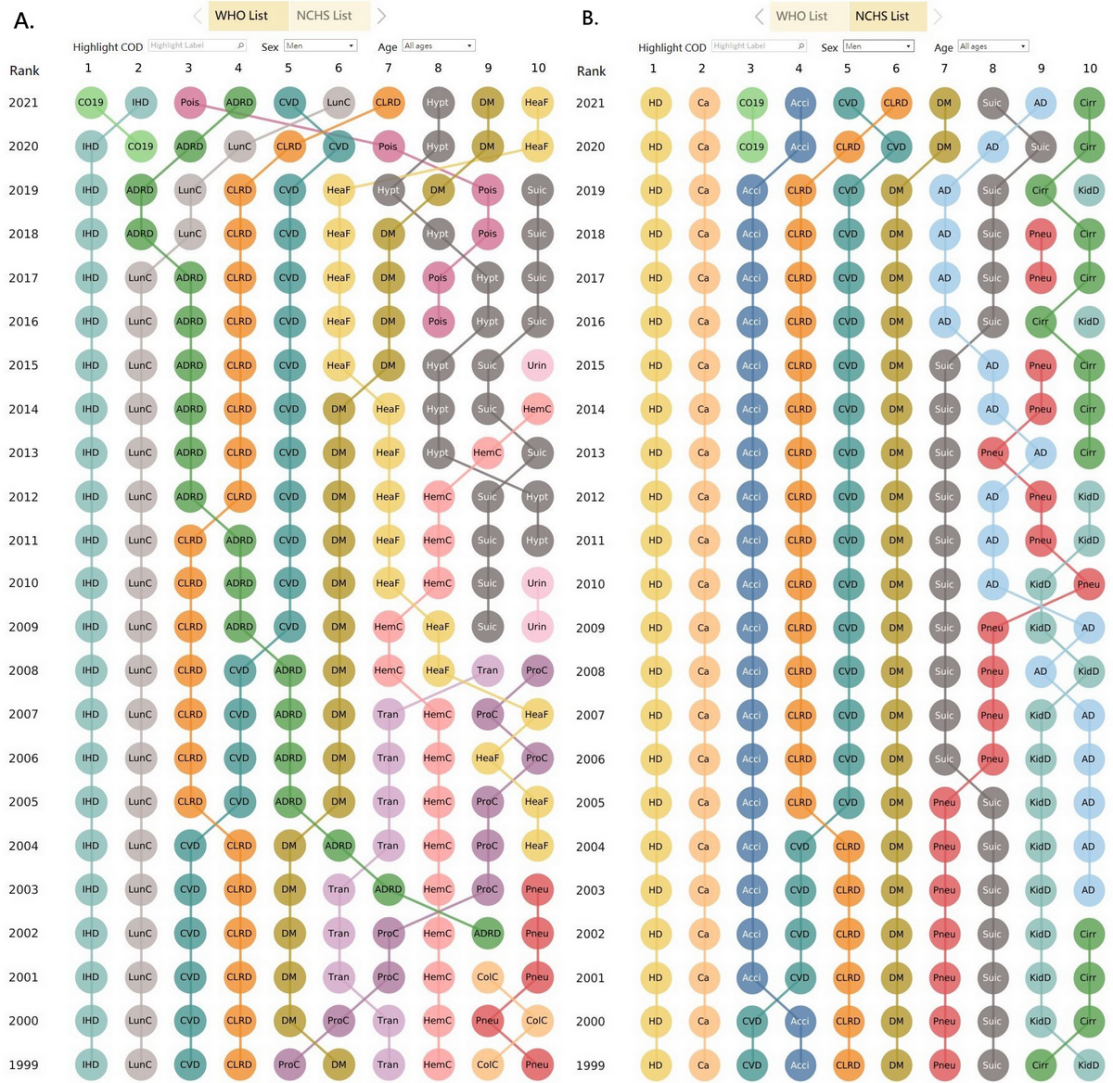
Ranking of the 10 leading causes of death for men of all ages in the United States according to (A) the World Health Organization (WHO) list and (B) the National Center for Health Statistics (NCHS) list. This dashboard is accessible [17]. Acci: accidents; AD: Alzheimer disease; ADRD: Alzheimer disease and related dementias; Ca: cancer; Cirr: chronic liver disease and cirrhosis; ColC: colon cancer; CLRD: chronic lower respiratory disease; CO19: COVID-19; CVD: cerebrovascular disease (stroke); DM: diabetes mellitus; HD: heart disease; HeaF: heart failure; HemC: hematopoietic cancer; Hypt: hypertensive diseases; IHD: ischemic heart disease; KidD: kidney diseases; LunC: lung cancer; Pneu: pneumonia; Pois: unintentional poisoning; ProC: prostate cancer; Suic: suicide; Tran: transportation accidents; Urin: urinary diseases.

The 10 leading CODs for men of all ages according to the WHO and NCHS lists are presented in Figure 2. We noted an abrupt increase in the rank of unintentional poisoning according to the WHO list, from ninth in 2019 to seventh in 2020 and third in 2021 (Figure 2A). In contrast, the accidents category was the third leading COD from 2001 to 2019 and the fourth leading COD in 2020 and 2021 (Figure 2B). Several CODs such as cirrhosis, kidney disease, and pneumonia, which appeared among the 10 leading CODs according to the NCHS list (Figure 2B), were squeezed out from the 10 leading CODs if the WHO list was used.

**Figure 3:**
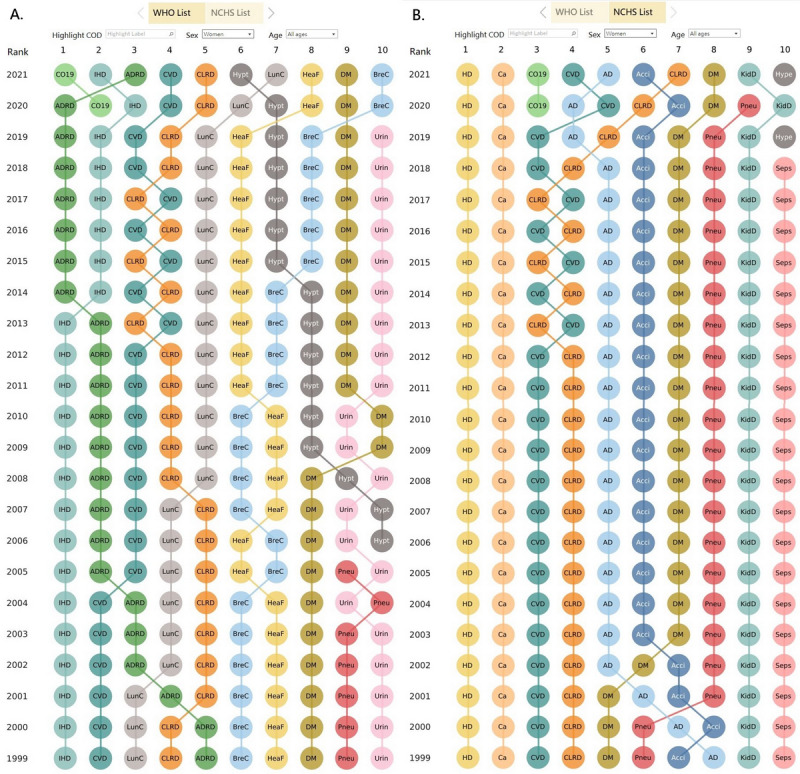
Ranking of the 10 leading causes of death for women of all ages in the United States according to (A) the World Health Organization (WHO) list and (B) the National Center for Health Statistics (NCHS) list. This dashboard is accessible [17]. Acci: accidents; AD: Alzheimer disease; ADRD: Alzheimer disease and related dementias; BreC: breast cancer; Ca: cancer; CLRD: chronic lower respiratory disease; CO19: COVID-19; CVD: cerebrovascular disease (stroke); DM: diabetes mellitus; HD: heart disease; HeaF: heart failure; Hypt: hypertensive diseases; IHD: ischemic heart disease; KidD: kidney diseases; LunC: lung cancer; Pneu: pneumonia; Seps: sepsis.

The rankings of leading CODs among women of all ages in the WHO and NCHS lists are compared in Figure 3. According to the WHO list, ADRD ranked first from 2014 to 2020 and third in 2021 (Figure 3A). However, the Alzheimer disease category used in the NCHS list ranked fifth from 2002 to 2018, fourth from 2019 to 2020, and fifth in 2021 (Figure 3B). According to the WHO list, hypertensive diseases persistently increased in ranking, from eighth in 2009-2014 to seventh in 2015-2020, and to sixth in 2021 (Figure 3A). However, the category “hypertension” used in the NCHS list did not appear in the 10 leading CODs during the study period, except in 2019 and 2021, when it was ranked tenth (Figure 3B). By contrast, several CODs including pneumonia, kidney disease, and sepsis that appeared among the 10 leading CODs according to the NCHS list did not appear among the 10 leading CODs if the WHO list was used.

**Figure 4:**
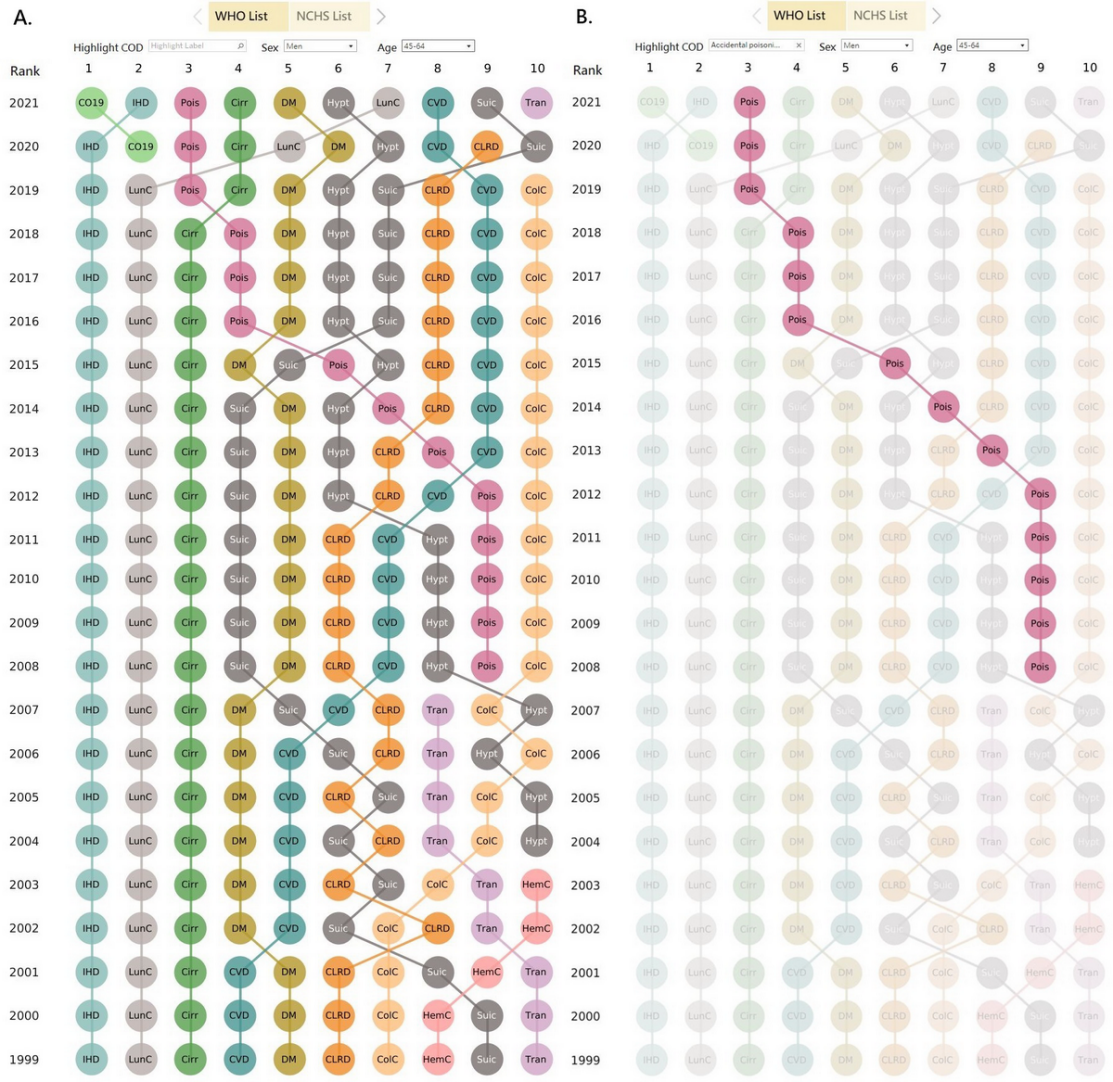
Ranking of 10 leading causes of death for men aged 45-64 years in the United States according to the World Health Organization (WHO) list (A) without using and (B) with using the highlight filter. This dashboard is accessible [17]. Cirr: chronic liver disease and cirrhosis; ColC: colon cancer; CLRD: chronic lower respiratory disease; CO19: COVID-19; CVD: cerebrovascular disease (stroke); DM: diabetes mellitus; HemC: hematopoietic cancer; Hypt: hypertensive diseases; IHD: ischemic heart disease; LunC: lung cancer; Pois: unintentional poisoning; Suic: suicide; Tran: transportation accidents.

Figure 4 contrasts the rankings of the 10 leading CODs among men aged 45-64 years according to the WHO list with and without using a highlight filter. For example, by selecting unintentional poisoning in the highlight filter, we can better visualize the marked increase in the rankings of unintentional poisoning (Figure 4B).

## Discussion

### Principal Findings

The findings of this study indicate that profiles of the rankings of the 10 leading CODs differ depending on which list is used. Several CODs were among the 10 leading CODs in several sex and age subgroups when the WHO list was used, including brain, breast, colon, hematopoietic, lung, pancreas, prostate, and uterus cancer (all classified as cancer on the NCHS list); unintentional transport injuries; poisoning; drowning; and falls (which were all classified as accidents on the NCHS list). ADRD and hypertensive diseases, which have more comprehensive definitions in the WHO list, were ranked higher in the WHO list than similar categories in the NCHS list. By contrast, some CODs that appeared among the 10 leading CODs according to the NCHS list, such as cirrhosis, kidney disease, sepsis, and pneumonia, were squeezed out of the 10 leading CODs according to the WHO list. Through the use of a dashboard with bump charts, users can select a list, stratify the data by age and sex, and use a highlight filter to visualize the changes in the rankings of a particular COD across years.

The main reason for the differences between the lists in the COD rankings was the number of categories used to rank CODs. The NCHS list had 52 categories, and the WHO list had 65 categories (Table S1 in Multimedia Appendix 1). Selecting which list to use involves a trade-off. Some new specific CODs that existed in the 10 leading CODs according to the WHO list could not be revealed if the NCHS list was used. On the contrary, some CODs that appeared in the 10 leading CODs according to the NCHS would be squeezed out if the WHO list were used. The use of a more comprehensive definition for ADRD and hypertensive diseases might sacrifice the specificity, meaning that some individuals with ADRD diagnosis might be false positives. To obtain the most comprehensive information for health policy decision-making, we recommend using both lists.

Several studies have examined the changes in the rankings of the leading CODs in the United States [18-20]. Because the categories in the NCHS list are relatively broad, prominent changes cannot be detected, except for accidents, which increased in rank from fifth in 2011 to third in 2018 [19]. The pattern of changes in the leading CODs in the United States according to the WHO list differed from that in Japan, Korea, and Taiwan [7]. According to the WHO list, the leading COD from 1999 to 2020 in the United States was IHD, whereas in Japan, Korea, and Taiwan, the leading COD in most of the years during this period was stroke [7].

### Strengths and Limitations

This study has several strengths. First, this study was the first to use the WHO list to illustrate changes in the ranking of the leading CODs in the United States. Using the WHO list enabled us to detect several drastic increases in the ranks of some leading CODs, which could not be revealed according to the NCHS list. The second strength of this study was the use of bump charts, which aid in visualizing the changes in rankings of specific CODs in a ladder-like manner. The third strength was the creation of a dashboard, which enables viewers to select the specific demographic group they wish to examine using either the NCHS or the WHO list (or another list can be added) and highlight the changes of a particular COD.

However, several limitations should be considered when interpreting the findings of this study. First, the changes in the relative position of each rank on the bump charts did not correspond to the extent of the changes in the proportions of total deaths accounted for by each category. For example, ADRD increased in ranking between 2013 and 2020 (from second to first) but accounted for fewer proportion of deaths in 2020 than it did in 2013 (10.9% vs 12.1%, respectively). Therefore, an increase (or decrease) in ranking does not indicate an increase (or decrease) in either burden or risk. Second, although the WHO list uses more specific categories compared to the NCHS list, several categories are still relatively broad. For example, unintentional poisoning comprises ten 3-digit ICD-10 codes (X40-X49) that each correspond to a different drug or chemical. Third, both ranking lists are limited in terms of detail and are not comprehensive, meaning that not all deaths can be categorized and ranked. The increase in the number of deaths in some emergent diseases within the residual category could not be detected using rankings of leading CODs.

### Conclusions

The rankings of the leading CODs constitute a highly popular health statistic used to convey relative mortality burdens and are widely used by media and health advocates. A dashboard with bump charts can be used to improve visualizing the changes in the rankings of the leading CODs according to the WHO and NCHS lists as well as the demographic characteristics; it can also help users make informed decisions regarding the most appropriate ranking list for their needs.

## Appendix

Multimedia Appendix 1 International Classification of Diseases, Tenth Revision codes for the World Health Organization ranking list mapped to the National Center for Health Statistics ranking list.

## Abbreviations

ADRD: Alzheimer disease and related dementias
COD: cause of death
ICD-10: International Classification of Diseases, Tenth Revision
IHD: ischemic heart disease
NCHS: National Center for Health Statistics
WHO: World Health Organization

## Data Availability

The data that support the findings of this study are publicly available [17].
