# Correction: Dashboard With Bump Charts to Visualize the Changes in the Rankings of Leading Causes of Death According to Two Lists: National Population-Based Time-Series Cross-Sectional Study

**DOI:** 10.2196/56014

**Published:** 2024-01-09

**Authors:** Shu-Yu Tai, Ying-Chen Chi, Yu-Wen Chien, Ichiro Kawachi, Tsung-Hsueh Lu

**Affiliations:** 1 Department of Family Medicine Kaohsiung Municipal Ta-Tung Hospital and Kaohsiung Medical University Hospital; School of Medicine, College of Medicine Kaohsiung Medical University Kaohsiung Taiwan; 2 Department of Healthcare Information and Management School of Health Technology Ming Chuan University Taoyuan Taiwan; 3 Department of Public Health College of Medicine National Cheng Kung University Tainan Taiwan; 4 Department of Social and Behavioral Sciences Harvard T.H. Chan School of Public Health Boston, MA United States

In “Dashboard With Bump Charts to Visualize the Changes in the Rankings of Leading Causes of Death According to Two Lists: National Population-Based Time-Series Cross-Sectional Study” (JMIR Public Health Surveill 2023;9(1):e42149) the authors noted one error.

In the originally published article, the first affiliation incorrectly appeared as:

Department of Family Medicine, Kaohsiung Municipal Ta-Tung Hospital, Kaohsiung, Taiwan.

This has been changed to the following:

Department of Family Medicine, Kaohsiung Municipal Ta-Tung Hospital and Kaohsiung Medical University Hospital; School of Medicine, College of Medicine, Kaohsiung Medical University, Kaohsiung, Taiwan.

The correction will appear in the online version of the paper on the JMIR Publications website on January 9, 2024, together with the publication of this correction notice. Because this was made after submission to PubMed, PubMed Central, and other full-text repositories, the corrected article has also been resubmitted to those repositories.

