# Supplementary material for: Dashboard With Bump Charts to Visualize the Changes in the Rankings of Leading Causes of Death According to Two Lists: National Population-Based Time-Series Cross-Sectional Study
Source: JMIR Public Health Surveill. 2023 Jun 27;9:e42149. doi: 10.2196/42149 (PMC10337380; doi:10.2196/42149)
Supplement: Multimedia Appendix 1 [file publichealth_v9i1e42149_app1.docx]

**Table S1.** ICD-10 codes for the World Health Organization (WHO) ranking list and mapped to the National Center for Health Statistics (NCHS) ranking list

| **No** | **WHO category for ranking (ICD-10 codes)** | **Match** | **No** | **NCHS category for ranking (ICD-10 codes)** |
| --- | --- | --- | --- | --- |
| LC-01 | Intestinal infectious diseases (A00–A09) | Partial | 1#* | Salmonella infections (A01–A02) |
|  |  |  | 2# | Shigellosis and amebiasis (A03,A06) |
|  |  |  | 3 | Certain other intestinal infections (A04,A07–A09) |
| LC-02 | Tuberculosis (A15–A19) | Partial | 4# | Respiratory tuberculosis (A16) |
|  |  |  | 5# | Other tuberculosis (A17–A19) |
| LC-03 | Vector-borne diseases and rabies (A20, A44, A75–A79, A82–A84, A85.2, A90–A96, A98.0–A98.2, A98.8, B50–B57) | Partial | 12# | Arthropod-borne viral encephalitis (A83–A84,A85.2) |
| LC-04 | Vaccine-preventable diseases (A33–A37, A80, B01, B05, B06, B15, B16, B17.0, B18.0, B18.1, B18.9, B19, B26) | Partial | 6# | Whooping cough (A37) |
|  |  |  | 11# | Acute poliomyelitis (A80) |
|  |  |  | 14# | Viral hepatitis (B15–B19) |
|  |  | No | 7# | Scarlet fever and erysipelas (A38,A46) |
| LC-05 | Meningitis (A39, A87, G00–G03) | Partial | 8# | Meningococcal infection (A39) |
|  |  |  | 46# | Meningitis (G00,G03) |
| LC-06 | Septicaemia (A40–A41) | Exact | 9# | Septicemia (A40–A41) |
|  |  | No | 10# | Syphilis (A50–A53) |
|  |  | No | 13# | Measles (B05) |
| LC-07 | Human immunodeficiency virus [HIV] disease (B20–B24) | Exact | 15# | Human immunodeficiency virus (HIV) disease (B20–B24) |
|  |  | No | 16# | Malaria (B50–B54) |
|  |  | No | 17 | Other and unspecified infectious and parasitic diseases and their sequelae (A00,A05,A20–A36,A42–A44,A48–A49,A54-A79,A81–A82,A85.0–A85.1,A85.8,A86–B04,B06–B09,B25–B49,B55–B99) |
|  |  | No | 18 | Malignant neoplasms of lip, oral cavity and pharynx (C00–C14) |
| LC-08 | Malignant neoplasm of esophagus (C15) | Exact | 19 | Malignant neoplasm of esophagus (C15) |
| LC-09 | Malignant neoplasm of stomach (C16) | Exact | 20 | Malignant neoplasm of stomach (C16) |
| LC-10 | Malignant neoplasm of colon, sigmoid, rectum and anus (C18–C21) | Exact | 21 | Malignant neoplasms of colon, rectum and anus (C18–C21) |
| LC-11 | Malignant neoplasm of liver and intrahepatic bile ducts (C22) | Exact | 22 | Malignant neoplasms of liver and intrahepatic bile ducts (C22) |
| LC-12 | Malignant neoplasm of gallbladder and other parts of biliary tract (C23–C24) | No |  |  |
| LC-13 | Malignant neoplasm of pancreas (C25) | Exact | 23 | Malignant neoplasm of pancreas (C25) |
| LC-14 | Malignant neoplasm of larynx (C32) | Exact | 24 | Malignant neoplasm of larynx (C32) |
| LC-15 | Malignant neoplasm of trachea, bronchus and lung (C33–C34) | Exact | 25 | Malignant neoplasms of trachea, bronchus and lung (C33–C34) |
| LC-16 | Melanoma and other malignant neoplasms of skin (C43–C44) | Partial | 26 | Malignant melanoma of skin (C43) |
| LC-17 | Malignant neoplasms of female breast (C50) | Exact | 27 | Malignant neoplasm of breast (C50) |
| LC-18 | Malignant neoplasm of uterus (C53–C55) | Exact | 28 | Malignant neoplasm of cervix uteri (C53) |
|  |  |  | 29 | Malignant neoplasms of corpus uteri and uterus, part unspecified(C54–C55) |
| LC-19 | Malignant neoplasm of ovary (C56) | Exact | 30 | Malignant neoplasm of ovary (C56) |
| LC-20 | Malignant neoplasm of prostate (C61) | Exact | 31 | Malignant neoplasm of prostate (C61) |
| LC-21 | Malignant neoplasm of kidney, except renal pelvis (C64) | Partial | 32 | Malignant neoplasms of kidney and renal pelvis (C64–C65) |
| LC-22 | Malignant neoplasm of bladder (C67) | Exact | 33 | Malignant neoplasm of bladder (C67) |
| LC-23 | Malignant neoplasm of brain (C71) | Partial | 34 | Malignant neoplasms of meninges, brain and other parts of central nervous system (C70–C72) |
| LC-24 | Malignant neoplasms of lymphoid, haematopoietic and related tissue (C81–C96) | Exact | 35 | Hodgkin’s disease (C81) |
|  |  |  | 36 | Non-Hodgkin’s lymphoma (C82–C85) |
|  |  |  | 37 | Leukemia (C91–C95) |
|  |  |  | 38 | Multiple myeloma and immunoproliferative neoplasms (C88,C90) |
|  |  |  | 39 | Other and unspecified malignant neoplasms of lymphoid, hematopoietic and related tissue (C96) |
|  |  | No | 40 | All other and unspecified malignant neoplasms (C17,C23–C24,C26–C31,C37–C41,C44–C49,C51–C52,C57-C60,C62-C63,C66,C68–C69,C73–C80,C97) |
| LC-25 | Benign neoplasms, in situ and uncertain behaviour (D00–D48) | Exact | 41# | In situ neoplasms, benign neoplasms and neoplasms of uncertain or unknown behavior (D00–D48) |
| LC-26 | Diabetes (E10–E14) | Exact | 43# | Diabetes mellitus (E10–E14) |
| LC-27 | Malnutrition and nutritional anaemias (D50–D53, E40–E64) | Partial | 42# | Anemias (D50–D64) |
|  |  |  | 44 | Malnutrition (E40–E46) |
|  |  |  | 45 | Other nutritional deficiencies (E50–E64) |
| LC-28 | Disorders of fluid, electrolyte and acid-based balance dehydration (E86–E87) | No |  |  |
| LC-29 | Dementia and Alzheimer disease (F01, F03, G30) | Partial | 48# | Alzheimer's disease (G30) |
| LC-30 | Mental and behavioural disorders due to psychoactive substance use (F10–F19) | No |  |  |
| LC-31 | Parkinson’s disease (G20) | Partial | 47# | Parkinson's disease (G20–G21) |
| LC-32 | Epilepsy and status epilepticus (G40–G41) | No |  |  |
| LC-33 | Chronic rheumatic heart diseases (I05–I09) | Partial | 49 | Acute rheumatic fever and chronic rheumatic heart diseases (I00–I09) |
| LC-34 | Hypertensive diseases (I10–I15) | Exact | 50 | Hypertensive heart disease (I11) |
|  |  |  | 51 | Hypertensive heart and renal disease (I13) |
|  |  |  | 60# | Essential (primary) hypertension and hypertensive renal disease (I10,I12) |
| LC-35 | Ischemic heart diseases (I20–I25) | Exact | 52 | Acute myocardial infarction (I21–I22) |
|  |  |  | 53 | Other acute ischemic heart diseases (I24) |
|  |  |  | 54 | Atherosclerotic cardiovascular disease, so described (I25.0) |
|  |  |  | 55 | All other forms of chronic ischemic heart disease (I20,I25.1–I25.9) |
| LC-36 | Pulmonary heart disease and diseases of pulmonary circulation (I26–I28) | Partial | 59 | All other forms of heart disease (I26–I28,I34–I38,I42–I49,I51) |
| LC-37 | Nonrheumatic valve disorders (I34–I38) |  |  |  |
| LC-38 | Cardiomyopathy (I42) |  |  |  |
| LC-39 | Cardiac arrest (I46) |  |  |  |
| LC-40 | Cardiac arrhythmias (I47-I49) |  |  |  |
|  |  | No | 56 | Acute and subacute endocarditis (I33) |
|  |  | No | 57 | Diseases of pericardium and acute myocarditis (I30–I31,I40) |
| LC-41 | Heart failure and complications and ill-defined heart disease (I50–I51) | Partial | 58 | Heart failure (I50) |
| LC-42 | Cerebrovascular diseases (I60-I69) | Exact | 61# | Cerebrovascular diseases (I60–I69) |
| LC-43 | Atherosclerosis (I70) | Exact | 62# | Atherosclerosis (I70) |
| LC-44 | Aortic aneurysm and dissection (I71) | Exact | 63# | Aortic aneurysm and dissection (I71) |
|  |  | No | 64 | Other diseases of arteries, arterioles and capillaries (I72-I78) |
|  |  | No | 65 | Other disorders of circulatory system (I80–I99) |
| LC-45 | Acute respiratory diseases other than influenza and pneumonia (J00–J06, J20–J22) | Partial | 68# 69 | Acute bronchitis and bronchiolitis (J20–J21) Unspecified acute lower respiratory infection (J22, U04) |
| LC-46 | Influenza and Pneumonia (J10-J18) | Exact | 66# | Influenza (J10–J11) |
|  |  |  | 67# | Pneumonia (J12–J18) |
| LC-47 | Chronic lower respiratory diseases (J40–J47) | Exact | 70# | Bronchitis, chronic and unspecified (J40–J42) |
|  |  |  | 71# | Emphysema (J43) |
|  |  |  | 72# | Asthma (J45–J46) |
|  |  |  | 73# | Other chronic lower respiratory diseases (J44,J47) |
|  |  | No | 74# | Pneumoconioses and chemical effects (J60–J66,J68) |
|  |  | No | 75 | Pneumonitis due to solids and liquids (J69) |
| LC-48 | Pulmonary oedema and other interstitial pulmonary diseases (J80–J84) | Partial | 76 | Other diseases of respiratory system (J00–J06,J30–J39,J67,J70–J98) |
| LC-49 | Respiratory failure (J96) |  |  |  |
|  |  | No | 77# | Peptic ulcer (K25–K28) |
| LC-50 | Appendicitis, hernia and intestinal obstruction (K35–K46, K56) | Partial | 78# | Diseases of appendix (K35–K38) |
|  |  |  | 79# | Hernia (K40–K46) |
| LC-51 | Cirrhosis and other diseases of liver (K70–K76) | Partial | 80# | Alcoholic liver disease (K70) |
|  |  |  | 81# | Other chronic liver disease and cirrhosis (K73–K74) |
|  |  | No | 82# | Cholelithiasis and other disorders of gallbladder (K80-K82) |
| LC-52 | Diseases of the musculoskeletal system and connective tissue (M00–M99) | No |  |  |
| LC-53 | Diseases of the urinary system (N00–N39) | Partial | 83 | Acute and rapidly progressive nephritic and nephrotic syndrome (N00–N01,N04) |
|  |  |  | 84 | Chronic glomerulonephritis, nephritis and nephritis not specified as acute or chronic and renal sclerosis unspecified (N02–N03,N05–N07,N26) |
|  |  |  | 85 | Renal failure (N17–N19) |
|  |  |  | 86 | Other disorders of kidney (N25,N27) |
|  |  |  | 87# | Infections of kidney (N10–N12,N13.6,N15.1) |
|  |  | No | 88# | Hyperplasia of prostate (N40) |
|  |  | No | 89# | Inflammatory diseases of female pelvic organs (N70–N76) |
| LC-54 | Pregnancy, childbirth and the puerperium (O00–O99) | Exact | 90# | Pregnancy with abortive outcome (O00–O07) |
|  |  |  | 91# | Other complications of pregnancy, childbirth and the puerperium (O10–O99) |
| LC-55 | Certain conditions originating in the perinatal period (P00–P96) | Exact | 92# | Certain conditions originating in the perinatal period (P00–P96) |
| LC-56 | Congenital malformations, deformations and chromosomal abnormalities (Q00–Q99) | Exact | 93# | Congenital malformations, deformations and chromosomal abnormalities (Q00–Q99) |
| LC-57 | Land transport accidents (V01–V89) | Partial | 96 | Motor vehicle accidents (V02-V04,V09.0,V09.2,V12-V14,V19.0-V19.2, V19.4-V19.6,V20-V79,V80.3-V80.5,V81.0-V81.1,V82.0-V82.1,V83–V86,V87.0–V87.8,V88.0–V88.8,V89.0,V89.2) |
|  |  |  | 97 | Other land transport accidents (V01,V05–V06,V09.1,V09.3–V09.9,V10–V11,V15–V18,V19.3,V19.8–V19.9,V80.0–V80.2, V80.6–V80.9,V81.2–V81.9,V82.2–V82.9,V87.9,V88.9,V89.1,V89.3, V89.9) |
|  |  | No | 98 | Water, air and space, and other and unspecified transport accidents and their sequelae (V90-V99,Y85) |
| LC-58 | Accidental falls (W00–W19) | Exact | 99 | Falls (W00-W19) |
| LC-59 | Nonintentional firearm discharge (W32–W34) | Exact | 100 | Accidental discharge of firearms (W32-W34) |
| LC-60 | Accidental drowning and submersion (W65–W74) | Exact | 101 | Accidental drowning and submersion (W65-W74) |
| LC-61 | Accidental threats to breathing (W75–W84) | No |  |  |
|  |  | No | 102 | Accidental exposure to smoke, fire and flames (X00-X09) |
| LC-62 | Accidental poisoning (X40–X49) | Exact | 103 | Accidental poisoning and exposure to noxious substances (X40-X49) |
|  |  | No | 104 | Other and unspecified nontransport accidents and their sequelae (W20-W31,W35-W64,W75-W99,X10-X39,X50-X59,Y86) |
| LC-63 | Intentional self-harm [suicide] (X60–X84) | Partial | 105 | Intentional self-harm (suicide) by discharge of firearms (X72-X74) |
|  |  |  | 106 | Intentional self-harm (suicide) by other and unspecified means and their sequelae (X60-X71,X75-X84,Y87.0) |
| LC-64 | Assault [homicide] (X85–Y09) | Partial | 107 | Assault (homicide) by discharge of firearms (X93-X95) |
|  |  |  | 108 | Assault (homicide) by other and unspecified means and their sequelae (X85-X92,X96-Y09,Y87.1) |
|  |  | No | 109# | Legal intervention (Y35,Y89.0) |
| LC-65 | Event of undetermined intent (Y10–Y34) | Partial | 110 | Discharge of firearms, undetermined intent (Y22-Y24) |
|  |  |  | 111 | Other and unspecified events of undetermined intent and their sequelae (Y10-Y21,Y25-Y34,Y87.2,Y89.9) |
|  |  | No | 112# | Operations of war and their sequelae (Y36,Y89.1) |
|  |  | No | 113# | Complications of medical and surgical care (Y40-Y84,Y88) |
| LC-99 | Symptoms, signs and ill-defined conditions (R00–R99) | Exact | 94 | Symptoms, signs and abnormal clinical and laboratory findings, not elsewhere classified (R00-R99) |
| LC-88 | Remainder (all the rest) | Partial | 95 | All other diseases (Residual) |
